# Maintenance of Species Boundaries Despite Ongoing Gene Flow in Ragworts

**DOI:** 10.1093/gbe/evw053

**Published:** 2016-03-14

**Authors:** Owen G. Osborne, Mark A. Chapman, Bruno Nevado, Dmitry A. Filatov

**Affiliations:** ^1^Department of Plant Sciences, University of Oxford, Oxford, United Kingdom; ^2^Department of Life Sciences, Imperial College London – Silwood Park Campus, Berkshire, United Kingdom; ^3^Centre for Biological Sciences, Faculty of Natural & Environmental Sciences, University of Southampton, Southampton, United Kingdom

**Keywords:** speciation with gene flow, hybridization, phylogenetic incongruence, clade diversification, introgression

## Abstract

The role of hybridization between diversifying species has been the focus of a huge amount of recent evolutionary research. While gene flow can prevent speciation or initiate species collapse, it can also generate new hybrid species. Similarly, while adaptive divergence can be wiped out by gene flow, new adaptive variation can be introduced via introgression. The relative frequency of these outcomes, and indeed the frequency of hybridization and introgression in general are largely unknown. One group of closely-related species with several documented cases of hybridization is the Mediterranean ragwort (genus: *Senecio*) species-complex. Examples of both polyploid and homoploid hybrid speciation are known in the clade, although their evolutionary relationships and the general frequency of introgressive hybridization among them remain unknown. Using a whole genome gene–space dataset comprising eight *Senecio* species we fully resolve the phylogeny of these species for the first time despite phylogenetic incongruence across the genome. Using a *D*-statistic approach, we demonstrate previously unknown cases of introgressive hybridization between multiple pairs of taxa across the species tree. This is an important step in establishing these species as a study system for diversification with gene flow, and suggests that introgressive hybridization may be a widespread and important process in plant evolution.

## Introduction

The “tree of life” has been one of the most enduring metaphors in evolutionary biology. The sole illustration in “On The Origin…” (Darwin 1859) depicts a model of species diversification in which speciation is bifurcating and irreversible. While Darwin’s concept stressed the importance of divergence by natural selection (Darwin 1859; Pinho and Hey 2010), the homogenizing effect that gene flow could play in preventing divergence was emphasized later, and many biologists have maintained that an external barrier to gene flow is necessary for speciation to occur (Dobzhansky 1935; Mayr 1963). It has long been known, however, that speciation without gene flow and tree-like evolution is an incomplete explanation of species diversification. Introgression and incomplete lineage sorting (ILS) result in different phylogenetic histories for different regions of a species’ genome (Pamilo and Nei 1988) and the hybrid origin of some taxa create reticulate nodes in the tree of life (Rieseberg 2006). Furthermore, it is becoming increasingly clear that species divergence can proceed without an initial external barrier to gene flow, and divergent selection may be sufficient to drive the process of divergence, with reproductive isolation coming much later (Rundle and Nosil 2005).

Introgression, the movement of genes from the gene pool of one species to another, through repeated hybridization and backcrossing (Anderson 1949), may be extremely prevalent in nature (and particularly in plants; Whitney et al. 2010). Interspecific hybridization and introgression has been detected in plants (Arnold et al. 1992; Strasburg and Rieseberg 2008; Muir et al. 2012), animals (Nevado et al. 2011; Pardo-Diaz et al. 2012; Fontaine et al. 2014), and many other groups of sexual organisms (Neafsey et al. 2010; Zardi et al. 2011; Sun et al. 2012). This is often the case despite divergent local adaptation and strong pre- and post-zygotic reproductive barriers (Lawton-Rauh et al. 2007; Chapman and Abbott 2010; Sambatti et al. 2012; Cui et al. 2013). Such introgressive hybridization, in addition to the more pervasive process of ILS, can cause significant phylogenetic incongruence between different genomic regions in a species complex and this can lead to difficulty in estimating the species phylogeny. Rather than being viewed simply as an inconvenience for phylogenetic inference, however, the various incongruent phylogenetic histories observed between loci should correctly be seen to represent a more accurate account of the history of a clade than any single bifurcating tree when hybridization has occurred (Rokas and Carroll 2006).

The fact that gene flow can, and may routinely, continue during and after species divergence, leads to the question of how species distinctness can be maintained in the face of such gene flow. Introgression can have a detrimental impact on the species involved, by homogenizing the regions of their genomes which have become divergently locally adapted, potentially leading to despeciation or the extinction of one taxon (Rhymer and Simberloff 1996; Webb et al. 2011). Conversely, it can be adaptive, by increasing variation within, and sharing globally adaptive mutations between, species and creating novel combinations of alleles (Seehausen 2004). The complex interactions of gene flow, drift, and selection; which may all vary spatially, temporally, and between loci; can combine to lead to diverse outcomes at the species level. Whether speciation will or will not occur, and to what extent species boundaries are maintained after initial divergence depends on these factors, and understanding the interaction of these processes during species diversification represents one of the most important challenges in evolutionary biology (Seehausen 2004; Nosil et al. 2009). What is clear is that interspecific gene flow is clearly prevalent in a wide range of taxa in which species integrity has not been completely compromised. Nevertheless, there are relatively few examples of studies investigating multiple species within a taxon, particularly with the high-throughput datasets needed to accurately represent the complex variation in phylogenetic signal which may be present throughout the genome. The relative importance of different processes in producing species diversity is yet to be determined and the “norm” of how a clade diversifies, and to what extent this differs between taxa, and why, is largely unknown.

To examine the frequency of introgression in plants, and begin to shed empirical light on the roles it might play, we undertook an analysis of eight diploid species of *Senecio. *This included six from the Mediterranean species complex (*S. aethnensis *Jan ex DC.*, S. chrysanthemifolius *Poir.*, S. leucanthemifolius *Poir., *S. gallicus *Vill.*, S. glaucus *L., and *S. vernalis* Waldst. & Kit.) and two outgroups (*S. madagascariensis *Poir. and *S. flavus *(Decne.) Sch. Bip.). The Mediterranean *Senecio *species complex provides a classic illustration of the diverse modes by which diversification can progress (Comes and Abbott 2001). It contains examples of a stable hybrid zone (Brennan et al. 2009), and both allopolyploid (Lowe and Abbott 1996, 2004; Kadereit et al. 2006; Pelser et al. 2012) and homoploid hybrid speciation (James and Abbott 2005). Therefore, many of the species are known to be capable of hybridizing and in addition to examples of hybrid speciation, some evidence for the more subtle process of introgressive hybridization has been found (Comes and Abbott 1999, 2001; Chapman and Abbott 2010), although this has received less attention (but see Coleman and Abbott 2003; Chapman and Abbott 2005, which discounted hypotheses of introgression in the group). Despite the possibility of hybridization between many of the species, they are highly phenotypically distinct and occur in a wide range of environments, including desert, alpine, steppe, rocky volcanic, and coastal Mediterranean habitats, so are likely to be divergently ecologically adapted. Thus, the system represents an opportunity to examine how clade divergence proceeds in the presence of gene flow. RNA-seq offers a cost-effective method to obtain large amounts of sequence data for protein-coding regions that arguably represent the most informative part of the genome. Furthermore, RNA-seq datasets already exist for three of our study species (Osborne et al. 2013). Thus, using a combination of previously published and new RNA-seq data, we (1) estimate the species-level evolutionary history of group, (2) investigate the extent of gene tree-species tree incongruence, which may have complicated previous phylogenetic and taxonomic analyses, and (3) detect past introgressive hybridization among the species. Our results shed light on the process of species diversification with gene flow and suggest that introgression has occurred with surprising frequency in the group.

## Materials and Methods

### Seed Collection, Plant Growth, and Sequencing

Plants were grown from wild-collected seed (locations shown in supplementary table S1, Supplementary Material online). Seeds were germinated on damp filter paper and seedlings were transferred to a soil/vermiculite mix in a growth room set at 19–21 °C with a 16-h photoperiod. To maximize the number of transcripts present, apical tissues were harvested from each plant (inflorescence, stem, and first apical leaf) when the first inflorescence opened, and frozen in liquid nitrogen. Tissue samples were ground while frozen and RNA was extracted with a Qiagen RNeasy plant kit (Qiagen, Crawley, UK) according to manufacturer’s instructions. The extraction procedure included an optional treatment with DNase (Qiagen). 3 µg of RNA per specimen was sent to the Wellcome Trust Centre for Human Genomics, Oxford (WTCHG) for sequencing. Paired-end libraries were prepared individually, barcoded, and then combined prior to sequencing. Libraries were sequenced in a single run using the Illumina Hiseq 2000 sequencing platform to produce 100 base-pair (bp) paired-end reads.

### Dataset Preparation

Base calling, adaptor trimming, and de-multiplexing of reads were undertaken as part of the WTCHG bioinformatics pipeline. This uses the native Illumina basecalling pipeline (Bustard 1.9) with default parameters. Raw reads for the *S. aethnensis, S. chrysanthemifolius*, and *S. vernalis *individuals used have already been published (Osborne et al. 2013) and are deposited in the Short Read Archive (SRA) under the accession number SRP028289. Reads for newly sequenced *S. leucanthemifolius, S. gallicus, S. glaucus, S. flavus,* and *S. madagascariensis *have also been deposited in the SRA under the accession SRP069830. Raw reads were imported into CLC Genomics Workbench 7 (CLC bio, Aarhus, Denmark; hereafter CLC). Reads were quality trimmed using an error probability cut-off parameter of 0.05 and a maximum of two ambiguous bases per read using the Modified Mott trimming algorithm in CLC (see CLC manual for details). Duplicate reads were removed using the CLC Duplicate Read Removal Plugin (raw read numbers and percentage of reads retained after trimming are reported in supplementary table S1, Supplementary Material online). *De novo *transcriptome assemblies of the two outgroup species (*S. madagascariensis* and *S. flavus*) were performed separately in CLC. We allowed the program to calculate an optimal k-mer length, which was 23 bp for both outgroup species. Further settings used were a minimum contig length of 300 bp, automatically determined maximum bubble size and scaffolding using paired end information (with mismatch cost of 2, insertion and deletion costs of 3, length fraction of 0.8, and similarity fraction of 0.95). To estimate transcriptome contiguity and quality, scaffolds were used as BLASTX (Altschul et al. 1990) queries against the *Arabidopsis thaliana *proteins (ftp://ftp.arabidopsis.org/home/tair/Proteins/, last accessed December 1, 2015) with default settings. Coverage of the top hit reference proteins was then used as a measure of transcript completeness.

To reduce the risk that results in downstream analyses could be biased by ancient introgression between the ingroup and outgroup lineages, we produced alignments using a reference-guided approach based on two outgroup *de novo* transcriptomes, those of *S. madagascariensis* and *S. flavus*. Trimmed reads from each individual were mapped on to the two outgroup reference transcriptomes separately using CLC with the following settings: length fraction = 0.8, similarity fraction = 0.9, automatic detection of paired end distances, mismatch cost = 2, insertion cost = 3, deletion cost = 3. Binary Alignment/Map format (BAM) files for each mapping were exported and the samtools/BCFtools package version 1.1 (Li et al. 2009) was used for variant calling and filtering. Each BAM file was used to produce a pileup file using samtools’ *mpileup* function with a base quality filter of 20 and a mapping quality filter of 20. The *bcftools call* command was then used for SNP calling with a minimum read depth filter of 8. Several further filters were implemented using the *bcftools filter* command: SNPs within three bases of indels, with a variant quality <10, and heterozygous SNPs with alleles represented by less than two reads were removed. The resulting Variant Call Format files were converted to fasta format using a custom C ++ script (available on request), indels were converted to missing data, and heterozygous SNPs were represented by IUPAC codes. Since two references, *S. madagascariensis* and *S. flavus*, were used for the mapping, two sets of alignments were produced and these were carried separately into downstream analyses.

### Phylogenetic Inference

Since ILS is likely to be widespread in recently diverged taxa such as the focal species, we estimated the phylogeny of the species using the multi-species coalescent-based approach of Mirarab et al. (2014), which accounts for ILS. First, for each reference-guided assembly, each single-scaffold alignment was used in a separate phylogenetic analysis using the GTRCAT model and 100 bootstrap replicates in RAxML 8 (Stamatakis 2014). The bootstrap replicates and best Maximum Likelihood (ML) trees from these analyses were then used to produce a species tree using ASTRAL (Mirarab et al. 2014) with 100 bootstrap replicates using both site-wise and gene-wise resampling. This produced a bootstrapped species tree for each of the two reference-guided datasets. As a secondary estimate of topology, and to determine branch lengths, we also undertook species tree estimation using a concatenation-based ML approach. Each of the two reference-guided assemblies, *S. flavus *and *S. madagascariensis*, were concatenated separately using a custom bash script (available on request). We then performed ML tree inference on these concatenated datasets in RAxML 8 using the GTRCAT model and 100 bootstrap replicates.

To visualize phylogenetic discordance between loci, we used the best ML tree for each contig from the per-locus RAxML analysis to produce DensiTree plots using Densitree version 2.2.1 (Bouckaert 2010). For each gene tree, nodes with bootstrap support <75% were collapsed using the *pruneTree *function in the *phangorn* package (Schliep 2011) in R 3.1.2 (R Core Development Team 2014). Gene trees with no nodes with over 75% bootstrap support were removed and each tree was rooted by *S. flavus *using the *root *function in the APE module (Paradis et al. 2004) in R. Rooted, pruned trees were then made ultrametric using the *chronos *function in the APE module (Paradis et al. 2004) in R with default settings. The resulting pruned, rooted, and ultrametric trees were then input into DensiTree (Bouckaert 2010). DensiTree plots were then produced using the consensus trees produced by DensiTree (in which branch lengths are averaged across all trees for a given topology) with the following settings (star tree, consensus width = 1, consensus intensity 28.1, and default values for all other settings). DensiTree plots were produced in this way for contigs for each of the two reference-guided assemblies combined.

To determine how much of the observed variation among gene trees was due to genuine incongruence, rather than simply lack of phylogenetic signal, we used Shimodaira–Hasegawa (SH) tests implemented in CONSEL version 0.2 on all contigs from each of the two reference-guided assemblies (Shimodaira and Hasegawa 1999, 2001). The procedure first uses the phylogenetic inference program PhyML version 3.0 (Guindon and Gascuel 2003) over two runs for each contig. The first run uses an unconstrained topology, and the second run constrains the topology to that of the presumed species tree obtained from the whole dataset (the topology from the coalescent-based and concatenation-based analyses for each of the two reference-based assemblies produced the same topology—see “Results” section). These were run using *GTR* substitution model without bootstrap replicates. Site-likelihoods from these runs were then compared in CONSEL and FDR correction was applied to the *P* values for each SH test (Benjamini and Hochberg 1995). Tests for which the FDR corrected SH-test *P* value (*Q* value) for the constrained tree is <0.05 are considered to significantly reject that species tree. The PhyML-Consel and per-contig RAxML pipelines were automated using custom perl and bash scripts (available on request).

### Tests for Introgression

To investigate possible introgression between the species as a cause of the observed incongruence, we first employed Patterson’s *D*-statistic test (Durand et al. 2011) which compares two phylogenetically incongruent site patterns of ancestral (A) and derived (B) alleles ABBA—(((A,B),B),A) and BABA—(((B,A),B),A) on a four-taxon phylogeny with the topology: (((Sp.1,Sp.2),Sp.3),Outgroup). If the incongruence is due to ILS, the frequencies of these site patterns are expected to be equal, but in the case of introgression between Sp.3 and either Sp.1 or Sp.2, they are expected to be skewed toward the site pattern that clusters the introgressing taxa together. Block Jackknifing (with each transcript representing a single block in the context of our dataset) was then used to determine significance. We used the *doAbbaBaba *function in ANGSD (Korneliussen et al. 2014) to test every phylogenetically congruent three-species subtree from the six European species using both *S. flavus* and *S. madagascariensis *as the outgroup/mapping-reference separately. This approach estimates counts of ABBA and BABA sites using base counts from BAM files applying a minimum read coverage filter of 5, and a minimum mapping quality filter of 20 to potential ABBA/BABA sites (Korneliussen et al. 2014). To minimize the effect of outgroup choice (and any bias caused by potential past introgression from the outgroup) we considered only the tests in which both outgroups produced similar results. All *P* values were corrected for multiple testing using the method of Benjamini and Hochberg (1995).

## Results

### Datasets

Our sequencing produced between 12,591,356 and 36,420,882 raw paired-end reads per species. After trimming, between 99.82% and 99.95% of reads were retained for further analysis (supplementary table S1, Supplementary Material online). Trimmed data from *S. flavus *was assembled into 25,035 contigs and *S. madagascariensis *was assembled into 29,739 contigs over 300 bp with respective N50 values of 1,102 and 1,093 bp and total assembly lengths of 23,420,882 and 25,857,323 bp (number of reads reported in supplementary table S1, Supplementary Material online). BLASTX searches against the *Arabidopsis *transcriptome were used to estimate transcript contiguity and transcriptome completeness, and both transcriptomes showed good coverage of the *Arabidopsis *proteins, indicating that transcript completeness was high (24.9% of *S. flavus *contigs and 24.3% of *S. madagascariensis *contigs aligned to at least 90% of their top hit, supplementary fig. S1, Supplementary Material online; 35.92% and 36.91% of *Arabidopsis *proteins were hit by the *S. flavus *and *S. madagascariensis *assemblies, respectively). Data from all eight species were mapped to the reference assemblies of the two outgroup species and, after filtering, the alignment based on the *S. flavus* reference contained 22,100 transcripts and the alignment based on the *S. madagascariensis* reference contained 25,431 transcripts. Effective (i.e., post-filtering) mapping coverage per species ranged from 32.75 to 73.64 to the *S. flavus *reference and from 33.60 to 70.17 to the *S. madagascariensis *reference (supplementary table S1, Supplementary Material online). The two reference-based datasets were carried separately into further analyses.

### Phylogenetic Inference

**Fig. 1.—:**
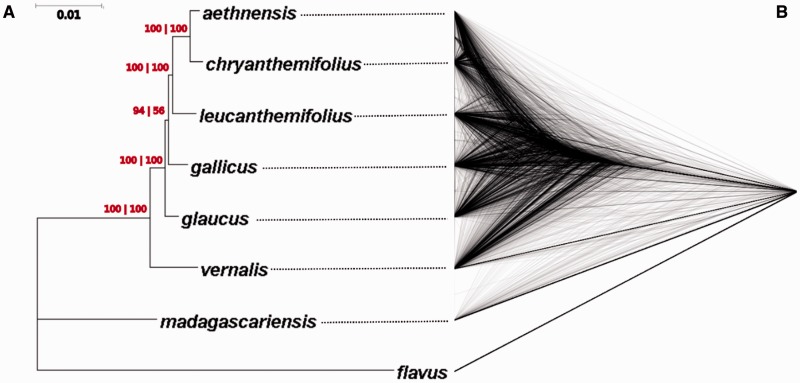
Phylogenetic reconstruction and gene tree-species tree incongruence. (*A*) Species phylogeny estimated using RAxML (Stamatakis 2014) and ASTRAL (Mirarab et al. 2014), both of which produced the same topology. Branch lengths are the mean of those produced using RAxML on both reference-based datasets. For each node, bootstrap support from the method of Mirarab et al. (2014) for the *S. flavus* and *S. madagascariensis* reference-based assemblies are shown before and after the bar, respectively. (*B*) A DensiTree plot of ML gene trees for all contigs. For each gene tree, nodes with <75% bootstrap support are collapsed and gene trees which subsequently contain more than two polytomies are excluded. For each unique topology among gene trees, branch lengths are averaged among all gene trees showing that topology. Results using trees from both reference-based assemblies combined are also shown.

Using both the multi-species coalescent-based method of Mirarab et al. (2014) and the concatenation-based ML method on both reference-based alignments produced the same topology (fig. 1*A*). To visualize the level of gene tree-species tree incongruence across the genome, gene trees for each transcript from both of the reference-based alignments (those used for estimation of the species tree with the multi-species coalescent-based approach, above) were used to build a DensiTree plot (Bouckaert 2010). When gene tree topologies are viewed in this way, they are clearly highly variable, but a sister relationship between *S. aethnensis *and *S. chrysanthemifolius* and monophyly of *S. aethnensis, S. chrysanthemifolius, S. leucanthemifolius, S. glaucus, S. gallicus*, and *S. vernalis *can clearly be seen (fig. 1*B*). The variation among gene tree topologies could be due to a lack of information in any single contig, or genuine incongruence, for example, from ILS or introgression. To determine whether data from individual contigs significantly rejected the inferred species tree, we implemented a series of SH tests to compare the fit of the estimated species tree and an unconstrained topology to the data. This showed that 1,561 (7.06%) of *S. flavus* reference-based contigs and 1,918 (7.54%) of *S. madagascariensis* reference-based contigs significantly reject the species tree (*P* < 0.05) which dropped to 370 and 582, respectively, after FDR correction.

### Detection of Introgression

The high level of gene tree-species tree incongruence could be due to either ILS or introgression. To differentiate between these possibilities we first used a *D*-statistic approach in which each species–tree–congruent triplet of ingroup species (once with each of the outgroups *S. madagascariensis* and *S. flavus* as mapping reference and outgroup used to polarize SNPs) was used in an ABBA–BABA test (Durand et al. 2011). The results indicated a large number of introgression events amongst the Mediterranean species had occurred, which were widely distributed across the tree (table 1). Assuming the species tree topology is correct, introgression was inferred in 12/20 tests using *S. flavus* as a reference, and 11/20 tests using *S. madagascariensis*. There was a high level of agreement between the results using each reference, with 10/20 being significant with both references, and 7/20 being non-significant in both, thus only three tests showed disagreement between the references (table 1). There was also a strong and highly significant correlation between the *D*-statistic estimates from the two references (Pearson’s product moment correlation test: *P *= 9.4 × 10^−^^8^, *R *= 0.895).

**Table 1:** ABBA–BABA Test Results

Test species			Mapping reference/outgroup			
			*S. flavus*		*S. madagascariensis*	
Sp. 1	Sp. 2	Sp. 3	*D*±SE	*Q*-value	*D*±SE	*Q*-value
chry	aeth	vern	−0.01±0.007	0.208	0.003±0.007	0.775
**gall**	aeth	**vern**	−0.027±0.006	<0.001	−0.053±0.006	<0.001
chry	aeth	gall	−0.001±0.006	0.937	0.001±0.006	0.937
**gall**	chry	**vern**	−0.014±0.006	0.036	−0.053±0.006	<0.001
leuc	aeth	gall	0.01±0.006	0.094	0.007±0.005	0.295
leuc	chry	gall	0.017±0.006	0.004	0.006±0.006	0.388
gall	leuc	vern	−0.004±0.006	0.547	−0.005±0.006	0.498
**leuc**	aeth	**vern**	−0.02±0.006	0.002	−0.035±0.006	<0.001
**chry**	aeth	**leuc**	−0.038±0.006	<0.001	−0.027±0.006	<0.001
leuc	chry	vern	−0.011±0.006	0.094	−0.044±0.006	<0.001
**glau**	aeth	**vern**	−0.044±0.006	<0.001	−0.054±0.006	<0.001
chry	aeth	glau	−0.013±0.006	0.067	−0.005±0.007	0.528
**glau**	chry	**vern**	−0.035±0.006	<0.001	−0.065±0.006	<0.001
**gall**	aeth	**glau**	−0.075±0.005	<0.001	−0.093±0.006	<0.001
**gall**	chry	**glau**	−0.071±0.006	<0.001	−0.096±0.005	<0.001
**gall**	leuc	**glau**	−0.074±0.005	<0.001	−0.088±0.005	<0.001
glau	gall	vern	−0.019±0.006	0.002	−0.01±0.006	0.129
leuc	aeth	glau	0±0.006	0.941	−0.004±0.005	0.523
leuc	chry	glau	0.009±0.006	0.129	−0.01±0.006	0.103
**glau**	leuc	**vern**	−0.021±0.006	<0.001	−0.017±0.006	0.007

Results are shown for each for all phylogenetically congruent triplets of species using both outgroups as the mapping reference and outgroup. Species abbreviations are as follows: aeth: *S. aethnensis*, chry: *S. chrysanthemifolius*, leuc: *S. leucanthemifolius*, gall: *S. gallicus*, glau: *S. glaucus*, vern: *S. vernalis*. Species inferred to have introgressed in tests using both outgroups are highlighted in bold.

## Discussion

### Species Level Phylogeny and Gene Tree–Species Tree Incongruence

Despite the large amount of work on the Mediterranean *Senecio* species complex (e.g., Lowe and Abbott 2004; Hegarty et al. 2006; Kim et al. 2008; Brennan et al. 2009; Chapman and Abbott 2010; Pelser et al. 2012) a fully resolved phylogenetic history of the species had previously remained elusive (Comes and Abbott 2001; Pelser et al. 2007). This is mirrored by long-standing difficulties experienced by taxonomists in species identification and establishing satisfactory species delimitations amongst the Mediterranean species complex (Crisp 1972; Alexander 1979). Our results may go some way to explaining these difficulties since a proportion of genes significantly rejected the inferred species tree. This incongruence could also clearly be seen when individual gene trees were combined into a DensiTree plot (fig. 1*B*). Since the species are recently diverged, and thus ILS is likely to be widespread in the species complex, using a method such as that of Mirarab et al. (2014) which takes ILS into account, is likely to produce a more accurate species tree topology than concatenation-based methods (Mirarab et al. 2014). Nevertheless, the method does not account for gene flow between the species. Interspecific gene flow can create false monophyletic relationships, as well as making inference of correct monophyletic relationships more likely when gene flow is between sister species (Leaché et al. 2014) and evidence of extensive gene flow between the pair of sister species in this study, *S. aethnensis *and *S. chrysanthemifolius, *has been previously reported (Chapman et al. 2013; Muir et al. 2013; Osborne et al. 2013). Therefore, the phylogenetic hypothesis presented here, as well as future phylogenetic efforts in these species using similar methods, should be taken with some level of caution.

Those caveats notwithstanding, the phylogeny we have inferred has important implications. Perhaps the most notable feature was the fact that *S. aethnensis *and *S. chrysanthemifolius *were sister species (with respect to the species samples in this study). This is important because *S. aethnensis *and *S. chrysanthemifolius *have been previously postulated to be a case of recent ecological speciation (Osborne et al. 2013). Both species have very limited geographical ranges which abut in an altitude-associated hybrid zone where they hybridize extensively. Nevertheless, they are highly phenotypically distinct and there is evidence for divergent selection between them and low levels of both pre- and post-zygotic reproductive isolations (Brennan et al. 2009, 2014; Ross 2010; Ross et al. 2012; Chapman et. al. 2016). Thus, their apparent monophyly, at least relative to the other species we have sampled in this study, supports the possibility that they may have speciated *in situ* as a result of their differential adaptation to high and low altitude habitats. The sister relationship should be taken with some caution however. First, since extensive enough introgression can cause the incorrect inference of sister relationships between species, and *S. aethnensis *and *S. chrysanthemifolius *are known to undergo introgressive hybridization, then it is possible that this sister relationship could be incorrect (Leaché et al. 2014). This scenario assumes such a high level of gene flow that genetic swamping of one species with the other has occurred (Kutschera et al. 2014; Leaché et al. 2014). However, since the phylogenetic support for this relationship is so strong, then what remains of the original recipient taxon today if this was the case represents a very small proportion of the genome. Thus, the species as they exist today are essentially sister species across the vast majority of their genomes. A more important point regarding the *S. aethnensis*–*S. chrysanthemifolius *sister relationship is that data were not available for all species in the clade. Previous analyses based on chloroplast DNA and allozymes have found that *S. rupestris*, a species found in mountainous regions of central and southern Europe clusters with *S. aethnensis* in phylogenies, although there was very low statistical support for this relationship (bootstrap support <50%; Abbott et al. 2002). Thus, a focus of future work should be to produce a high-throughput phylogenetic analysis of the clade including multiple accessions of all species in the Mediterranean *Senecio *species complex, particularly *S. rupestris*, to confirm or deny the sister relationship between *S. aethnensis *and *S. chrysanthemifolius. *It is also worth noting that the only node with bootstrap support <100% was that partitioning *S. glaucus *from the clade containing *S. gallicus, S. leucanthemifolius, S. chrysanthemifolius*, and *S. aethnensis. *The phylogenetic positions of *S. glaucus, S. gallicus *and *S. leucanthemifolius *are also the most difficult to discern when viewing the *DensiTree *plot. These three species are all widespread species with partially overlapping ranges. It is possible that more frequent episodes of hybridization between the more widely distributed species in the clade could have extensively muddied the phylogenetic waters in Mediterranean *Senecio*. Indeed, there is strong evidence for introgression between *S. gallicus *and *S. glaucus *from the ABBA–BABA tests.

### Introgression Is Widespread in the Group

Gene tree–species tree incongruence can have many sources, which can be broadly divided into coalescent processes: the incomplete sorting of ancestral variation; and reticulate processes: which include introgressive hybridization, hybrid speciation, and vector-mediated horizontal gene transfer. Here, we provide evidence that at least part of the explanation for the high levels of gene tree–species tree incongruence identified is extensive introgressive hybridization in the clade.

Our system of multiple ABBA–BABA tests can provide some insight with respect to the phylogenetic position of introgression events, although the exact phylogenetic position and timing of introgression can often not be inferred. For example, assuming the species–tree topology is correct, multiple tests support introgression between *S. vernalis *and each of *S. leucanthemifolius, S. gallicus*, and *S. glaucus*. This result could be interpreted in several ways. First, it could represent separate episodes of introgression between *S. vernalis *and each of these species. Second, it could result from a more ancient introgression event between the ancestor of *S. vernalis *and the common ancestor of *S. leucanthemifolius, S. gallicus, S. glaucus, S. aethnensis*, and *S. chrysanthemifolius *with introgressed material subsequently being lost in *S. aethnensis *and *S. chrysanthemifolius*. And third, it could be due to introgression between *S. vernalis* and only one of the three species: with the remaining significant tests in this case resulting from either subsequent introgression between the recipient species and the other two, or to introgression into *S. vernalis* of genetic polymorphisms shared by *S. leucanthemifolius, S. gallicus*, and *S. glaucus*. Furthermore, these tests do not preclude introgression between *S. vernalis *and *S. aethnensis/chrysanthemifolius*. This could still have occurred, but if so, it occurred to a greater extent between *S. vernalis *and the other three species in the clade. A final caution regarding the interpretation of ABBA–BABA tests is that ancestral population structure could potentially provide false-positive results. It has been shown that some very specific cases of population structure can give rise to *D-*statistic patterns which are indistinguishable from introgression (see Durand et al. 2011; Eriksson and Manica 2012) although this seems an unlikely source of such a large number of significant tests. Overall, while the interpretation of multiple ABBA–BABA tests can be ambiguous in terms of the exact phylogenetic position of introgression it is clear that introgression has been widespread in the clade.

In a genus with such widespread interspecific hybridization, finding an outgroup with no previous contact with the focal species is challenging. The lineages containing both *S. madagascariensis *and *S. flavus *may have experienced historical hybridization with the clade containing our focal species (Kadereit et al. 2006; Pelser et al. 2012). This is potentially problematic because the *D*-statistic approach used could be affected by unknown introgression from the outgroup used to polarize genetic variation because this would introduce uncertainty of the ancestral state (Durand et al. 2011). For this reason, we used two species which were not closely related to each other (Pelser et al. 2007) for our *de novo* reference transcriptomes and outgroups for these tests. The high level of agreement between the outgroup replicates is encouraging: 17 out of 20 ABBA–BABA tests were either significant with both outgroups, or neither. We only considered tests which were significant in both after multiple test correction as evidence for introgression. Similarly, there was a very strong and significant correlation between the results using each outgroup in the *D*-statistic values provided by the ABBA–BABA tests. The genus *Senecio *has experienced a large number of known hybrid speciation events (Comes and Abbott 1999; Kadereit et al. 2006; Pelser et al. 2012) and it is possible that this is common throughout the genus, so it would be challenging to find outgroups for which no introgression since the split with the focal species could be guaranteed with any certainty. Our results underline the fact that such problems can be ameliorated, and a higher level of confidence in tests of introgression can be reached, by using multiple outgroups.

**Fig. 2.—:**
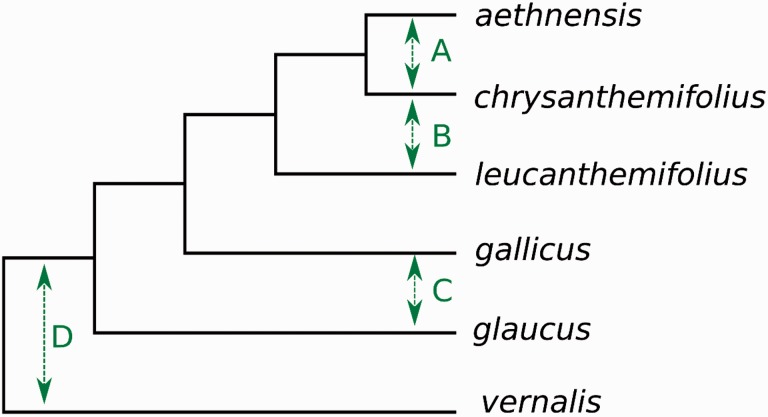
The scenario explaining the results of introgression analyses in this article as well as those in previous study (Osborne et al. 2013) which requires the fewest number of episodes of introgression. Green arrows represent introgression events and letters refer to table 2. Branch lengths are arbitrary. See table 2 for justification.

One of our specific conclusions matched those reached in previous work: that of gene exchange between *S. glaucus *and *S. vernalis*. A secondary contact zone has been reported between *S. glaucus *and *S. vernalis* in Israel (Comes and Abbott 1999) in which introgression has been inferred from sharing of cpDNA haplotypes between the species despite an ITS phylogeny placing them in distinct well supported clades. The *S. glaucus *and *S. vernalis *accessions used in this article were from geographically distant populations (Morocco and Cyprus, respectively; supplementary table S1, Supplementary Material online) suggesting that sharing of introgressed genetic material between the species is not restricted to contemporary parapatric populations in the Near East. The most parsimonious interpretation (i.e., the scenario which requires the fewest number of episodes of introgression) is reported in figure 2 and table 2. We do not assert, however, that this is necessarily the most likely scenario. Indeed, it is quite possible that the history of introgression in the clade is far more complex than this, and involves multiple episodes of introgression between multiple lineages or consistent low-level introgression throughout their evolution.

**Table 2:** Possible Interpretations of Results from Introgression Analyses

Hypothesis	Most parsimonious interpretation	Evidence from this study	Alternative interpretations	Evidence from previous studies
A	Introgression between the *S. aethnensis* and *S. chrysanthemifolius* lineages	n/a	n/a	Well documented in the literature. See e.g. Brennan et al. (2009)
B	Introgression between the *S. leucanthemifolius* and *S. chrysanthemifolius* lineages. Including introgression of variation shared by *S. aethnensis and S. chrysanthemifolius* into *S. leucanthemifolius*	An excess of shared incongruent SNPs between *S. leucanthemifolius* and *S. chrysanthemifolius* relative to *S. aethnensis*	n/a	Not previously reported
C	Introgression between *S. gallicus* and *S. glaucus*	An excess of shared incongruent SNPs between *S. gallicus* and *S. glaucus* relative to *S. leucanthemifolius, S. chrysanthemifolius* and *S. aethnensis*	Incorrect tree topology, since the node uniting *S. gallicus, S. leucanthemifolius, S. chrysanthemifolius* and *S. aethnensis* had low bootstrap support	Not previously reported
D	Introgression between the *S. vernalis* lineage and the common ancestor of all other species in the clade, subsequent loss of introgressed variation in *S. aethnensis* and *S. chrysanthemifolius*	An excess of shared incongruent SNPs between *S. vernalis* and *S. gallicus* and *S. glaucus* relative to *S. aethnensis* and *S. chrysanthemifolius.* An excess of shared incongruent SNPs between *S. vernalis* and *S. glaucus* relative to *S. leucanthemifolius.* An excess of shared incongruent SNPs between *S. vernalis* and *S. leucanthemifolius* relative to *S. aethnensis*	Separate introgression between *S. vernalis* and *S. leucanthemifolius, S. gallicus* and *S. glaucus.* Introgression of *S. vernalis* alleles into *one of the species* and subsequent introgression of that material into the others. While the scenario presented in the “most parsimonious interpretation” column requires the fewest episodes of introgression, the requirement that the introgressed material is subsequently lost in *S. aethnensis* and *S. chrysanthemifolius* makes this scenario seem somewhat less plausible than independent introgression events between *S. vernalis* and *S. leucanthemifolius, S. glaucus* and *S. gallicus*	Evidence of introgression between *S. vernalis* and *S. glaucus* (Comes and Abbott 1999).

### Conclusions and Future Work

In this study, we have shed light on the process of species diversification in the presence of gene flow. Overall, we conclude that, despite their phenotypic differences, probable local adaptation and habitat preference differences, the clade as a whole has experienced widespread gene flow throughout a substantial portion of its evolutionary history. Indeed, every species examined was found to have exchanged genetic material with at least one other species when the results of this article and previous study (Chapman et al. 2013; Muir et al. 2013; Osborne et al. 2013) are considered. What largely remains to be seen is the evolutionary role introgression plays in the species, whether introgression has had major consequences for adaptation in the species complex, and which genomic regions are likely to have been involved in introgression. Unfortunately, this dataset is unsuited to identifying the specific loci which have introgressed between the species since the *D*-statistic is likely to be dominated by stochastic variation in the short regions of sequence data produced by RNA-seq (Martin et al. 2015). However, the completion of the *Senecio *Genome Project (T. Batstone, B Nevado, M.A. Chapman, O.G. Osborne, D.A. Filatov, R.J. Abbott, and S.J. Hiscock, in preparation) will give access to longer genomic windows, which could be used for this purpose with additional resequencing of the species investigated here. This would allow questions regarding the role of gene flow to be addressed in greater detail. The results presented here, finding as they do far more widespread introgression than was previously known in this clade, are an important step toward establishing the Mediterranean *Senecio *complex as one of the foremost systems in which to study the evolutionary consequences of gene flow during species diversification and suggests that introgressive hybridization may be a widespread and important process in plant evolution.

## Supplementary Material

Supplementary figure S1 and table S1 are available at *Genome Biology and Evolution* online (http://www.gbe.oxfordjournals.org/).

Supplementary Data
