# Supplementary material for: Maintenance of Species Boundaries Despite Ongoing Gene Flow in Ragworts
Source: Genome Biol Evol. 2016 Mar 14;8(4):1038–47. doi: 10.1093/gbe/evw053 (PMC4860686; doi:10.1093/gbe/evw053)
Supplement: Supplementary Data [file supp_8_4_1038__index.html]

Maintenance of Species Boundaries Despite Ongoing Gene Flow in Ragworts — Supplementary Data 

# Maintenance of Species Boundaries Despite Ongoing Gene Flow in Ragworts

## Supplementary Data

files

- Supplementary Data - docx file
