# Supplementary material for: Maintenance of Species Boundaries Despite Ongoing Gene Flow in Ragworts
Source: Genome Biol Evol. 2016 Mar 14;8(4):1038–47. doi: 10.1093/gbe/evw053 (PMC4860686; doi:10.1093/gbe/evw053)
Supplement: Supplementary Data [file supp_evw053_supplementary_resubmission_2.docx]

Figure S1. Coverage of the top blastx hit in the *Arabidopsis* proteome by *de novo* assembled contigs of *S. flavus* and *S. madagascariensis*

**
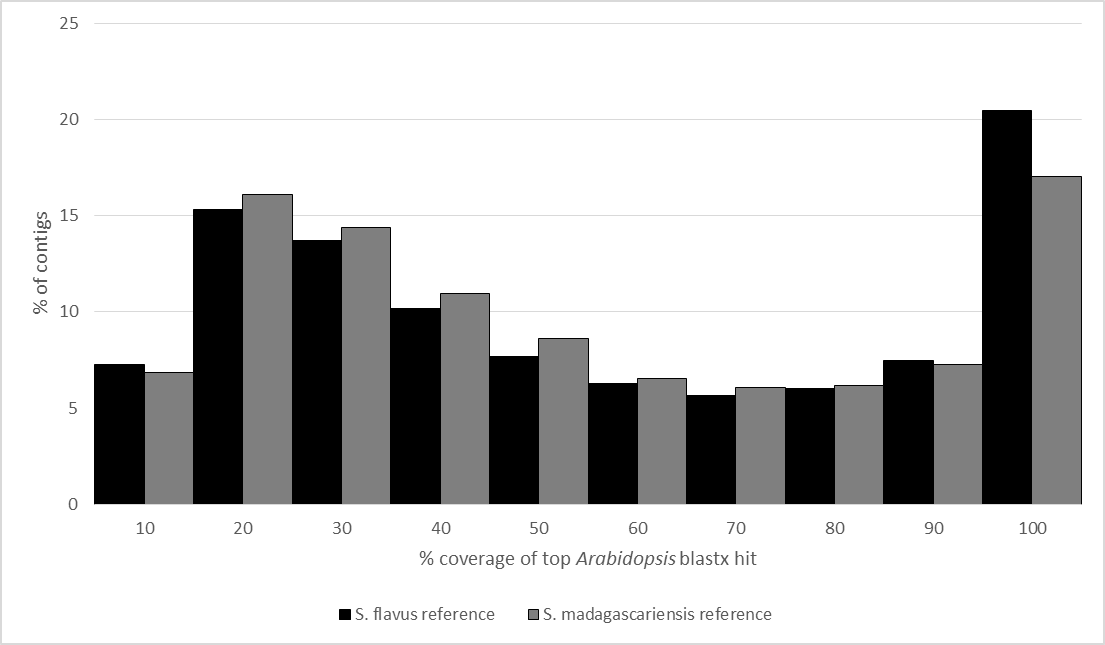
**

Table S1. Sampling location and number of reads for each species

| **Species** | **Location** | **N raw reads** | **Percentage of reads kept after trimming** | **Mean read depth ± SD (*S. flavus* – based alignments)** | **Mean read depth ± SD (*S. madagascariensis* – based alignments)** |
| --- | --- | --- | --- | --- | --- |
| *Senecio aethnensis* | Mount Etna, Sicily, Italy | 15,154,686 | 99.82% | 37.05 ± 194.30 | 35.82 ± 186.41 |
| *Senecio chrysanthemifolius* | Randozzo, Sicily, Italy | 12,591,356 | 99.83% | 35.34 ± 175.08 | 33.60 ± 166.14 |
| *Senecio flavus* | Puerto de la Peña, Fuerteventura, Canary Islands, Spain | 32,262,295 | 99.95% | 72.03 ± 293.20 | 70.17 ± 302.75 |
| *Senecio gallicus* | Amoreira, Algarve, Portugal | 19,038,966 | 99.61% | 32.75 ± 158.16 | 30.47 ± 149.18 |
| *Senecio glaucus* | Morocco | 31,234,638 | 99.92% | 60.98 ± 227.69 | 56.55 ± 211.54 |
| *Senecio leucanthemifolius* | Propriano, Corsica, France | 33,151,358 | 99.92% | 73.64 ± 250.56 | 69.18 ± 235.85 |
| *Senecio madagascariensis* | Kwazulu-Natal, South Africa | 34,583,848 | 99.87% | 70.82 ± 310.54 | 63.49 ± 287.16 |
| *Senecio vernalis* | Cyprus | 36,420,882 | 99.91% | 70.84 ± 255.06 | 65.98 ± 236.15 |

|  |
| --- |
|  |
